# Synthesis and characterization of fluorescent amino acid dimethylaminoacridonylalanine

**DOI:** 10.24820/ark.5550190.p011.498

**Published:** 2021-04-05

**Authors:** Chloe M. Jones, George A. Petersson, E. James Petersson

**Affiliations:** a Department of Chemistry; University of Pennsylvania; 231 South 34th Street; Philadelphia, Pennsylvania 19104-6323, USA; b Biochemistry and Molecular Biophysics Graduate Group; University of Pennsylvania; 3700 Hamilton Walk, Philadelphia, PA 19104, USA; c Temple University Institute for Computational Molecular Science, 1925 N. 12th Street, Philadelphia, PA 19122, USA

**Keywords:** Fluorescence, unnatural amino acid, acridone, twisted intramolecular charge transfer

## Abstract

Fluorescent amino acids are powerful biophysical tools as they can be used in structural or imaging studies of a given protein without significantly perturbing its native fold or function. Here, we have synthesized and characterized 7-(dimethylamino)acridon-2-ylalanine (Dad), a red-shifted derivative of the genetically-incorporable amino acid, acridon-2-ylalanine. Alkylation increases the quantum yield and fluorescence lifetime of Dad relative to a previously published amino acid, 7-aminoacridon-2-ylalanine (Aad). These properties of Dad make it a potentially valuable protein label, and we have performed initial testing of its ability to be genetically incorporated using an evolved aminoacyl tRNA synthetase.

## Graphical Abstract

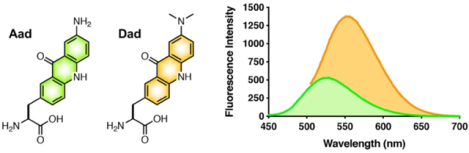

## Introduction

**Figure 1.**
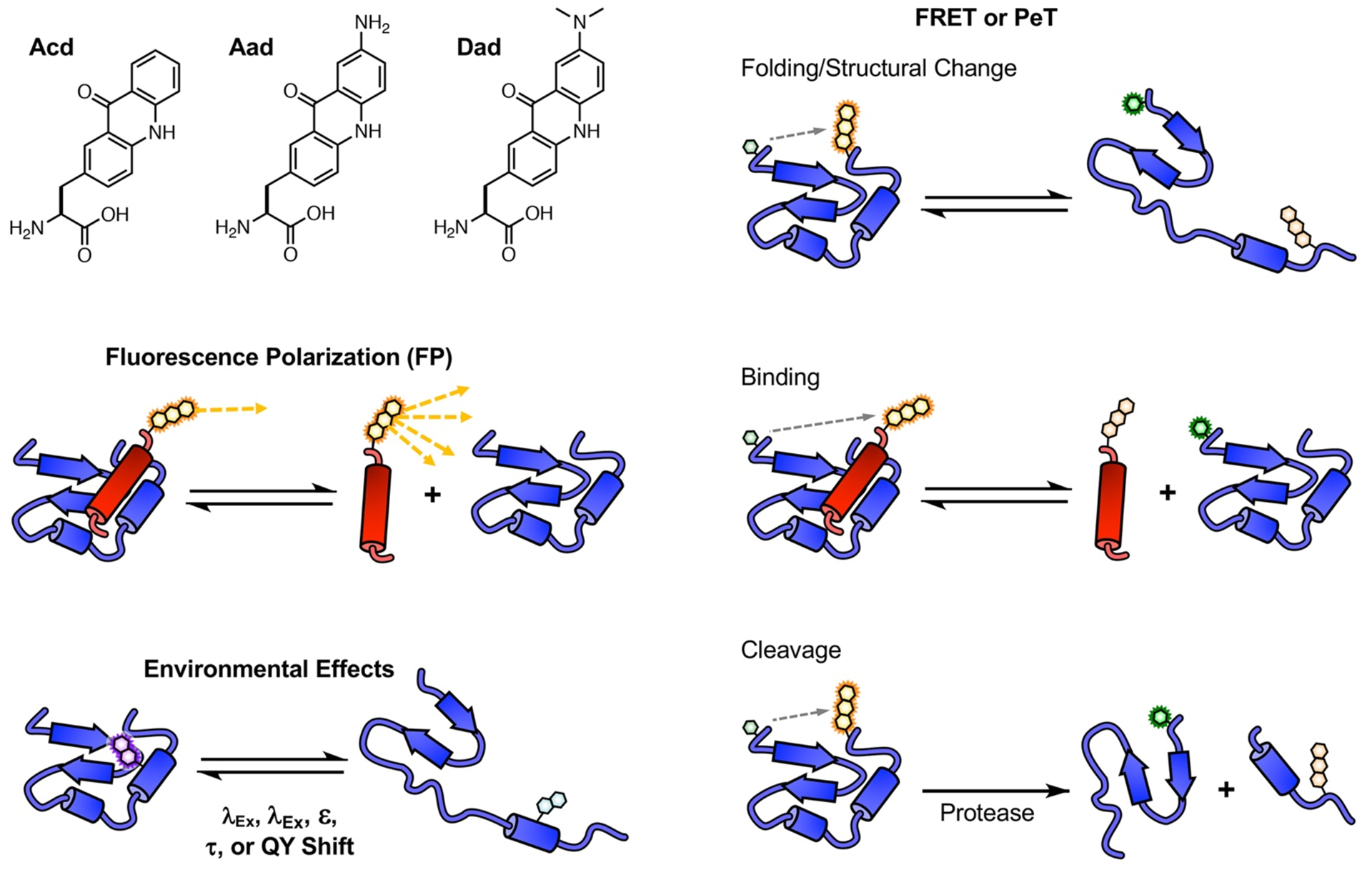
Protein biophysics experiments enabled by fluorescent Uaas.

Small fluorescent molecules can be designed to provide real-time information on a variety of biological processes, including the structure, function, and localization of proteins.^1–3^ Fluorescent unnatural amino acids (Uaas) are particularly valuable as they provide a minimally perturbing way to label proteins without disturbing their nascent structure and function.^4–6^ Proteins labeled with fluorescent Uaas can be used in microscopy experiments as well as a variety of biophysical experiments (Fig. 1). These include fluorescence polarization (FP) assays, which monitor changes in the rotational dynamics of the fluorophore, where polarization of the emitted light typically correlates inversely with molecular size.^7–8^ FP is often used to measure the binding of a smaller peptide or protein to a larger protein to determine binding rates or affinities. Environmental effects on fluorescent Uaa emission can be used to study conformational changes of a protein or binding to other biomolecules provided that they result in a change in the fluorophore environment.^9^ The effects can include changes in absorption and/or emission wavelength (λ), extinction coefficient (ε), fluorescence lifetime (τ), or quantum yield (QY) due to solvatochromic effects or quenching by nearby amino acids such as Tyr, Trp, Cys, or His. When proteins are labeled by two probes, distance changes can be studied based on interactions of the fluorescent Uaa with an appropriately matched partner through either Förster resonance energy transfer (FRET) or quenching due to photo-induced electron transfer (PeT).^10^ FRET requires spectral overlap of an acceptor chromophore with the donor fluorophore and PeT requires matching of donor and acceptor redox potentials. Both FRET and PeT can be used to monitor structural changes, binding events, and cleavage by proteases.

We have previously demonstrated the utility of acridon-2-ylalainne (Acd, Fig. 1), a genetically incorporable fluorophore with a blue emission.^11–18^ Acd’s high QY and long fluorescence lifetime can be valuable for biophysical studies, particularly FP where the range of protein sizes that can effectively be sensed increases with fluorophore lifetime.^19^ However, Acd’s UV excitation and blue emission can lead to photodamage.^20^ We have demonstrated that derivatizing Acd can red-shift its excitation and emission,^14^ which we hope to further develop towards a genetically incorporable Acd derivative FRET pair. However, our best candidate to date, 7-aminoacridon-2-ylalanine (Aad, Fig. 1) has a low fluorescence QY.^14^ We hypothesized that Aad’s loss in QY relative to Acd could be due to the amino group undergoing twisted intermolecular charge transfer (TICT) upon excitation.^21^ It has been shown that alkylation of pendant amines can reduce TICT through steric effects, increasing QY.^22–23^ Here, we have synthesize an alkylated Aad derivative, 7-(dimethylamino)acridon-2-ylalanine (Dad, Fig. 1). We characterize its fluorescent properties and evaluate its prospects for genetic incorporation using evolved aminoacyl tRNA synthetase (aaRS) enzymes.

## Results and Discussion

Dad was synthesized in five steps from tyrosine methyl ester and 2,5-aminobenzonitrile, using a route similar to the route previously employed to synthesize Acd and Aad. The key step was selective methylation of the 5-amino group of 2,5-aminobenzonitrile, which was achieved using Eschweiler-Clarke methylation in 49% yield. Since there are two amines that can be readily methylated, this step relies on the precise addition of 2.5 equivalents of paraformaldehyde followed by 3.0 equivalents of sodium cyanoborohydride. While the major product of this reaction is the desired doubly methylated species, the crude mixture also contained 6% of singly methylated material and 0.06% of triply methylated material according to liquid chromatography mass spectrometry (LC-MS) analysis. Successful methylation to form 2-amino-5-(dimethylamino)benzonitrile (**1**) was confirmed by examination of the aromatic peaks in the ^1^H NMR spectrum. If methylation instead occurred at the 2-amino group, the combination of substituent effects would result in the proton at the 3 position being more shielded than the proton at the 6 position. We instead see that the protons at carbons 3 and 6 are overlapping at ~6.8 ppm, consistent with previous NMR characterization of **1** by Harris *et al*.^24^ They synthesized **1** by reaction of dimethylamine with 5-chloro-2-nitrobenzonitrile to form 5-(dimethylamino)-2-nitrobenzonitrile, followed by tin reduction to form **1** without ambiguity as to the position of the dimethylamino group. Notably, our yield of 49%, while modest, is an improvement over their 39% yield over two steps.

Amino acid intermediate **3** was formed by Buchwald-Hartwig cross-coupling of **1** with *tert*-butyloxycarbonyl (Boc) protected, triflated tyrosine methyl ester **2** using BrettPhos Pd G1 and cesium carbonate. Compound **2** was synthesized from tyrosine *O*-methyl ester as previously described.^25^ Selective arylation of the 2-amino group of **1** was made possible by the prior methylation of the 5-amino group. Two alternative catalysts were also tested for this cross coupling step (RuPhos Pd G4 39%, *rac*-BINAP+ Pd(OAc)_2_ 10%), but were abandoned in favor of BrettPhos Pd G1 since they produced lower yields. The final Dad product (**4**) was formed by removal of the Boc and methyl ester protecting groups as well as Friedel-Crafts cyclization to form the acridone ring system by heating in sulfuric acid. After purification by reverse-phase chromatography, Dad was obtained in 52% yield (11% overall yield from 2,5-aminobenzonitrile).

### Spectroscopic characterization of Dad and comparison to Aad.

**Figure 2.**
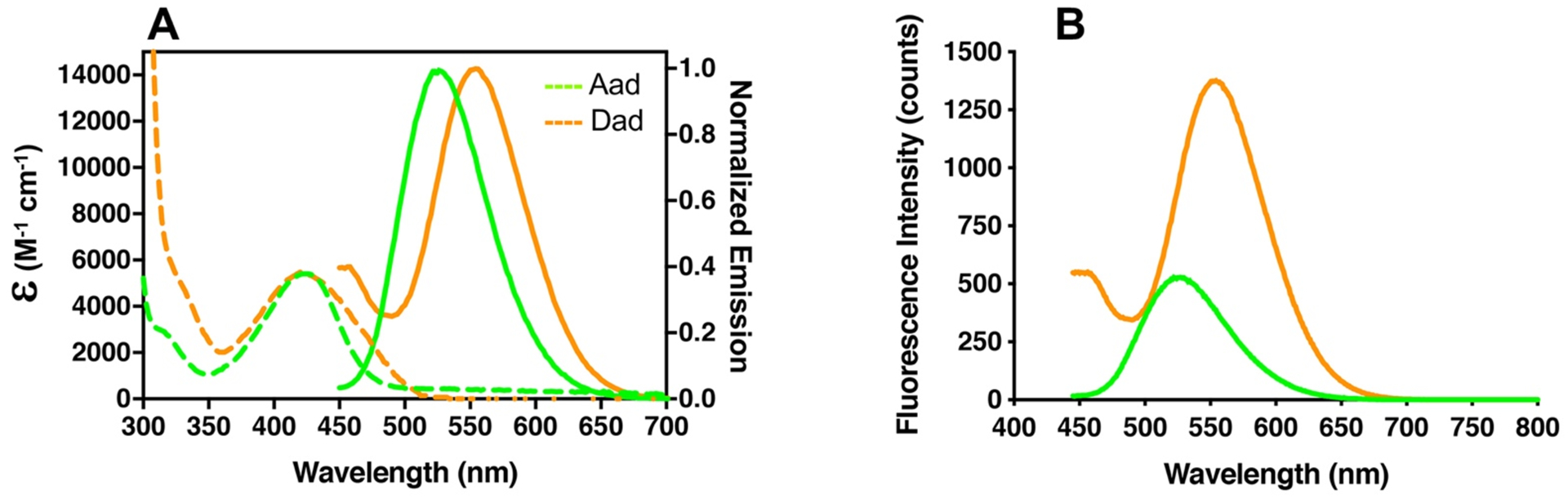
Comparison of Dad and Aad fluorescence properties. A) Comparison of excitation and emission for Aad and Dad in 50:50 CH_3_CN/PBS (50 μM). The emission spectra have been normalized to show the red-shift in λ_Em_ clearly. B) Comparison of Aad and Dad emission in 50:50 CH_3_CN/PBS (50μM). (λ_Ex_ 425 nm).

Following Dad synthesis, we measured its absorbance spectrum, extinction coefficient (ε), emission spectrum, fluorescence lifetime (τ), and QY (Fig. 2, Table 1, Fig. S1–6, and Table 2). These measurements were performed in a variety of solvents, including buffered aqueous solutions of varying pH. Dad has a broad absorption centered at 430 nm and emission maximum of 572 nm in a 50:50 mixture of acetonitrile and phosphate-buffered saline (PBS), pH 7.4 (Fig. 2). These values are red-shifted from the respective maxima for Aad at 422 nm and 530 nm. There is also a second Dad emission peak seen between 450 and 500 nm which comes from a protonated species (see below). The extinction coefficients of the Dad and Aad chromophores (Dad’ and Aad’, Fig. 4) were measured by serial dilution (Fig. S1). For Dad, ε_425_ 5415 ± 59 M^−1^•cm^−1^, which is similar to the extinction coefficient of Aad at 425 nm (ε_425_ 5409 ± 64 M^−1^•cm^−1^). An integrating sphere was used to measure QYs. The QYs of Dad and Aad are 0.25 and 0.10, respectively, in 50:50 CH_3_CN/PBS, making Dad 2.5-fold brighter than Aad (Table 1). The fluorescence lifetimes of Dad and Aad, measured by time correlated single photon counting (TCSPC), were similar (τ(Dad) 12.9 ns, τ(Aad) 11.8 ns) in 50:50 CH_3_CN/PBS (Table 2). In pure PBS, the lifetimes of both fluorophores are reduced (τ(Dad) 6.3 ns, τ(Aad) 6.7 ns) to values significantly smaller than the lifetime of Acd, which is 14.7 ns in buffer. The increased brightness and red-shift in emission should make Dad a superior probe to Aad for *in vitro* biophysical studies as well as for imaging.

**Figure 3.**
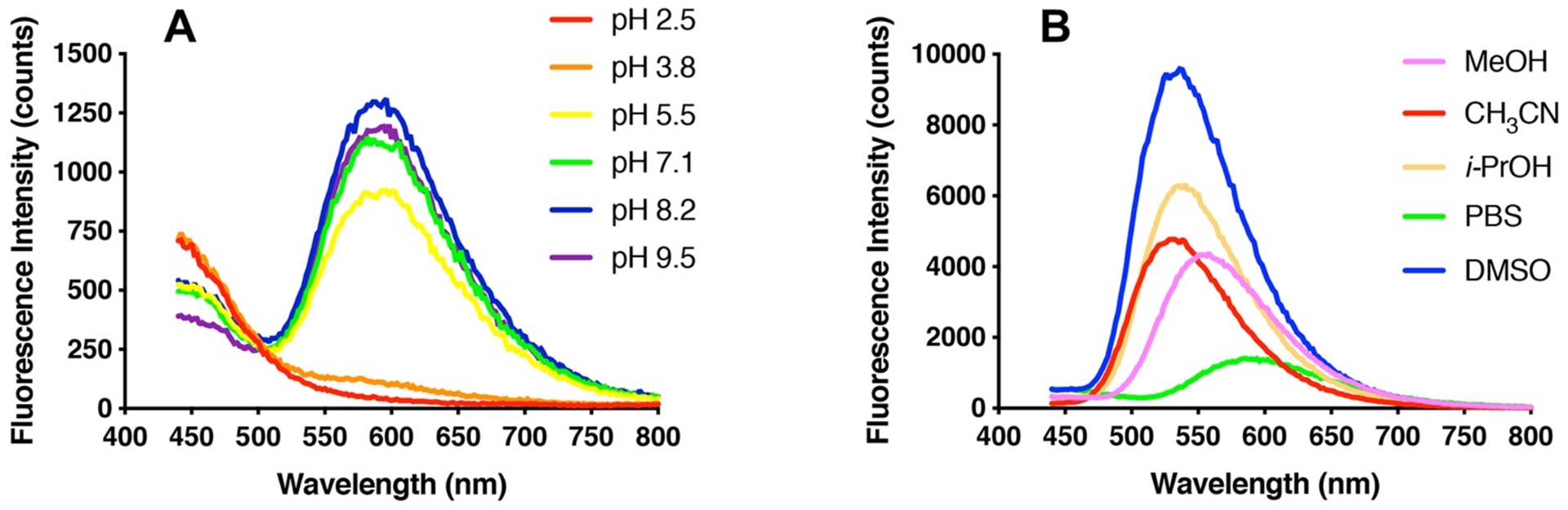
Solvent effects on Dad fluorescence. A. Emission spectra of 5 μM Dad in CBTP buffer at various pHs (λ_Ex_ 425 nm). B. Emission spectra of 5 μM Dad in various solvents (λ_Ex_ 425 nm).

Acd and Aad are known to exhibit solvatochromic effects that primarily alter the QY, but have minor effects on the shape of the emission spectrum, with the exception that the Aad emission spectrum changes dramatically at low pH, blue-shifting to resemble the Acd emission spectrum. This is because protonation of the amino group changes its hybridization to prevent it from extending the acridone π system. Similarly, measurement of the Dad emission spectrum in 50:50 mixtures of acetonitrile with buffers of varying pH shows that the long wavelength emission disappears as the pH drops below 4, with double emission peaks at 405 and 420 nm like Acd and Aad at low pH (Fig. 3 and Fig. S2). In organic solvents, Dad emission also blue-shifts, although not as dramatically, with a maximum around 550 nm that varies only slightly in buffer, isopropanol, methanol, acetonitrile, and DMSO (Fig. 3).

Dad QY similarly varies in different solvents, reaching a maximum of 0.57 in DMSO. In all organic solvents, the Dad QY is higher than that of Aad (Table 1 and Figs. S3–S4). The lifetime of Dad increases significantly in organic solvent relative to aqueous buffer, reaching a maximum of 19.91 ns in DMSO. In contrast, the lifetime of Aad in DMSO is 10.33 ns, although it is relatively similar to the lifetime of Dad in other solvents. These properties can be beneficial to Dad’s applications as a biophysical probe, where changes in emission maximum, QY, or lifetime can be used to monitor changes in protein conformation that would place Dad in environments of different polarity and/or sequester it from water.

The long lifetime of Dad in DMSO, nearly twice that of Aad, is surprising since the lifetimes of the two fluorophores are similar in other solvents (Table 2 and Figs. S5–S7). We suspected that this could result from aggregation of Dad in DMSO, but we observed no concentration dependence as the Dad lifetime remains unchanged over a 10 nM – 1 mM concentration range (Fig. S6). Another possibility is that the viscosity of DMSO hinders rotation of the dimethylamino group in Dad, further reducing TICT to give Dad a longer lifetime and higher QY in DMSO. These phenomena are commonly observed in fluorophores for which bond rotation contributes to electronic excitation, including TICT processes involving dimethylamino groups.^21, 26–28^ While further investigation of the photophysical mechanism of this effect is warranted, we should be able to harness this effect to study conformational change in proteins based on placing Dad in a more sterically restricted environment.

### Electronic structure calculations.

**Figure 4.**
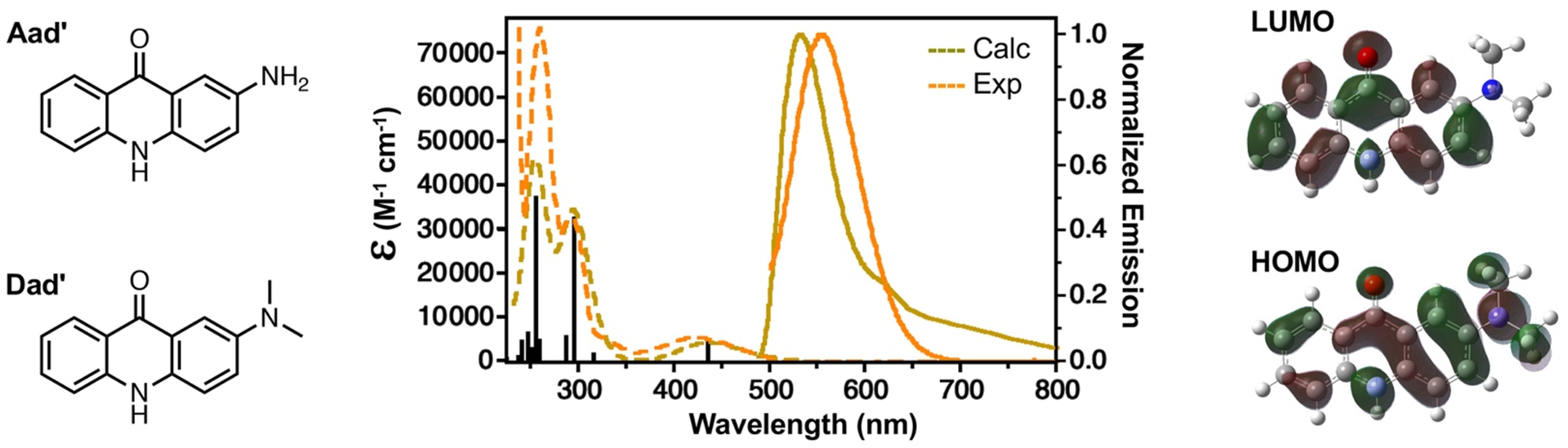
Comparison of experimental and computed Dad spectra. Experimental absorption and emission spectra for Dad were determined in 50:50 CH_3_CN/PBS. Computed spectra were determined from Dad’ APF-D/6–311+G(2d,p) vertical excitation and emission calculations and the shifted Aad’ vibronic emission spectrum. Excitation spectra are shown as dashed lines, emission spectra are shown as solid lines. Individual calculated state-to-state transitions are shown as black vertical lines.

We have previously performed *ab initio* electronic structure calculations of the absorption and emission spectra for 11 acridone derivatives, including 2-aminoacridone (Aad’, Fig. 4), the parent chromophore of Aad.^14^ We saw an excellent correlation (an average difference of 2.4% for all absorption and emission maxima) between calculated and observed values across this series, so we here wished to apply the same computational methods to 2-(dimethylamino)acridone (Dad’, Fig. 4), the parent chromophore of Dad. The calculations were performed using Gaussian16^™^ with an APF-D density functional and a 6–311+G(2d,p) basis set.^29^ The calculated vertical excitations and emissions for Dad’ (λ_Ex_ 436 nm, λ_Em_ 499 nm) are at lower energy than the corresponding transitions for Aad’ spectra (λ_Ex_ 406 nm, λ_Em_ 477 nm), matching the experimental observations of a 20–30 nm red-shift in the maxima. In our previous study of acridone derivatives, we found that Franck-Condon integrals from vibrational calculations could be used to generate spectra that matched the experimental solution phase emission spectra extremely well.^30^ Unfortunately, in this case, we found that differences in the ground and excited state geometries of prevented us from using the Franck-Condon strategy for Dad’. Therefore, we used the differences in the Dad’ and Aad’ vertical transitions to shift the Aad’ vibronic emission spectrum to approximate the Dad’ spectrum in water, matching the 50:50 CH_3_CN/PBS experimental data well (Fig. 4). Examination of the highest occupied molecular orbital (HOMO) and lowest unoccupied molecular orbital (LUMO) for Aad’ (Fig. S8) and Dad’ (Fig. 4) shows that the relevant transition for both molecules occurs by a shift in electron density from the pendant amino group into the acridone ring system, supporting our hypothesis that modulating TICT will improve brightness for these fluorophores. The ability to accurately model the excitation and emission of Dad will be valuable to designing derivatives that further red-shift its emission or improve its brightness.

### Dad incorporation for future protein studies.

**Figure 5.**
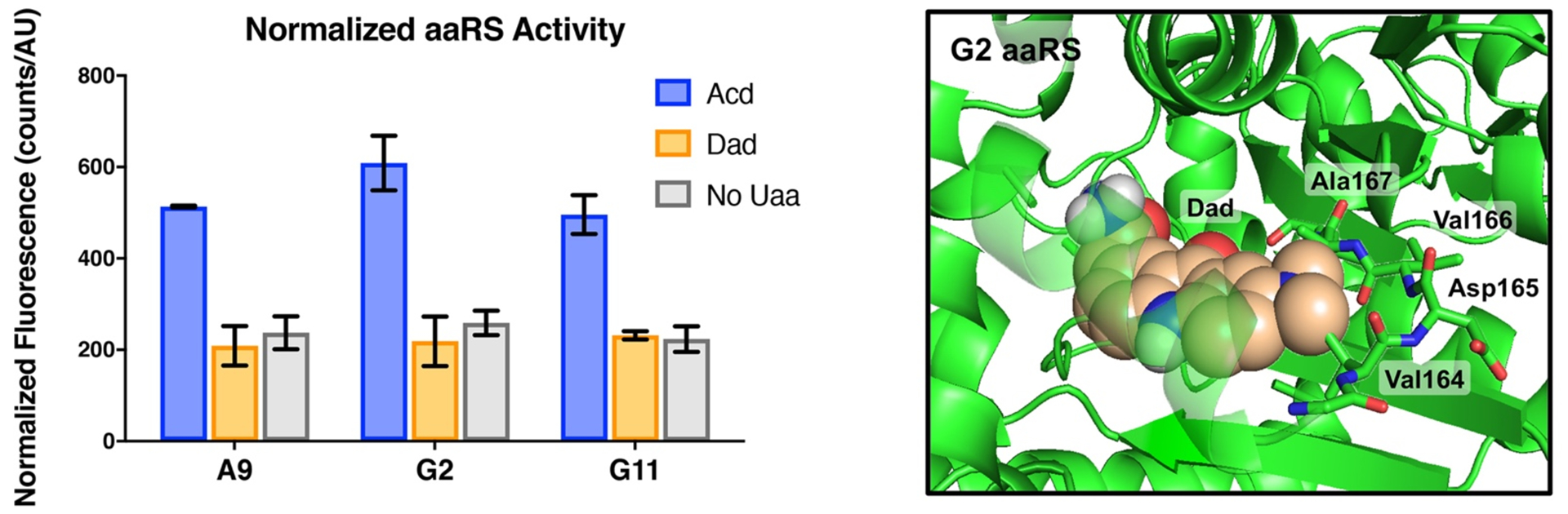
Prospects for genetic incorporation of Dad. Left: Incorporation efficiency of three *Mj* aaRS variants measured by sfGFP fluorescence intensity, normalized by cell density. None show significant Dad incorporation. Right: G2 aaRS active site structure (PDB ID: 4PBR)^31^ with Dad docked. Amino acids expected to interact with the dimethylamino group that are targets for mutation to improve Dad activity are highlighted.

While Dad can be readily incorporated into peptides in a Boc or fluorenylmethoxycarbonyl (Fmoc) protected form, our ultimate goal is to genetically incorporate Dad into proteins using Uaa mutagenesis, also known as amber suppression. This technique requires that an aaRS be evolved to specifically charge its cognate tRNA with the desired amino acid. The amino acid will then be incorporated into the growing protein chain in response to an amber (TAG) stop codon within the mRNA sequence. An initial screening method for potential aaRSs is to test whether they can charge the Uaa onto a tRNA for incorporation into super-folder green fluorescent protein (sfGFP) that contains an amber stop codon. Only successful incorporation will lead to full length sfGFP and therefore sfGFP fluorescence can be used as a readout for aaRS activation of the Uaa. We used three engineered aaRSs derived from *Methanocaldococcus jannachii* (*Mj*) tyrosyl tRNA synthetase which have been previously shown to incorporate Acd: A9, G2, and G11.^13^ G2 has also been shown to activate Aad. Our initial sfGFP assay results show little Dad incorporation by any of the aaRSs, with sfGFP fluorescence comparable to the negative control experiment with no amino acid added (Fig. 5). In contrast, sfGPF expression using Acd is robust for all three aaRS, as expected. It should be noted that although Acd and Dad are both fluorescent amino acids, since they are excited at 385 and 425 nm, respectively, they do not interfere with the sfGFP assay which uses excitation at 488 nm. Thus, it appears additional engineering of aaRSs will be necessary to genetically incorporate Dad.

Presumably the lack of aaRS activity toward Dad is a result of steric clashes with the dimethylamino group. Since a crystal structure is available for the G2 aaRS,^31^ and reliable homology models can be made for closely related mutants, computational modeling of Dad in the G2 active site can be used to identify the residues responsible for these clashes.^13^ Figure 5 shows Dad docked into the G2 aaRS active site by alignment to our previous model of docked Acd. Mutation of the highlighted residues Val164-Ala167, particularly Val164 and Ala167, could generate more permissive aaRSs to accommodate the additional steric bulk of Dad.^13^ These rationally designed efforts, as well as screening of random mutant libraries, are currently under way. If we can successfully generate an efficient aaRS for Dad, we will be able to easily incorporate it into proteins to take advantage of its utility as a small, green wavelength fluorophore for experiments like FP, as well as a potential FRET partner for Acd.

## Conclusions

We were able to modulate the fluorescence of an acridone-based scaffold through simple alkylation, leading to the concise synthesis of a new fluorescent amino acid. Our synthetic route relied on preferentially methylating the 5-amino group of 2,5-aminobenzonitrile to form 2-amino-5-(dimethylamino)benzonitrile (**1**) before cross-coupling and cyclization to form Dad in 11% overall yield. The alkylated Dad has a higher QY and longer fluorescence lifetime than Aad, increasing its value for biophysical studies like FRET and FP as well as for imaging studies. Dad can be protected with a Boc or Fmoc group and incorporated into peptides using solid phase synthesis methods. Ultimately, we would like to genetically incorporate Dad and will work to obtain mutants of the G2 aaRS that can charge Dad onto tRNA in order to express Dad-labeled proteins in *E. coli*. For imaging applications, pyrolysyl aaRSs from archea can be evolved for incorporation of Uaas in both *E. coli* and mammalian cells,^32^ and efforts are underway in our laboratory to engineer these aaRSs to activate Acd, Aad, and Dad.

## Experimental Section

### Synthesis of 2-amino-5-(dimethylamino)benzonitrile (1).

2,5 diaminobenzonitrile (500 mg, 3.78 mmol, 1 equiv.) and paraformaldehyde (284 mg 9.46 mmol, 2.5 equiv.) were stirred in glacial acetic acid (250 mL degassed with argon) for fifteen minutes. Sodium cyanoborohydride (713 mg, 11.35 mmol, 3 equiv.) was added to the reaction mixture and stirred at room temperature under argon atmosphere for approximately 16 hours. The reaction was quenched in an ice bath by adding 10 M sodium hydroxide dropwise until the mixture reached a pH of 6. This can also be seen as a color change from pink/orange to yellow. The crude material was extracted with ethyl acetate, washed with 0.1 M sodium hydroxide, dried with Na_2_SO_4_, and concentrated *in vacuo*. The single, double, and triply methylated species could not be distinguished using normal phase thin layer chromatography (TLC) and were instead followed by liquid chromatography-mass spectrometry (LC-MS). The crude mixture was purified by reverse phase chromatography (5% to 100% water with 0.1% TFA) in acetonitrile with 0.1% TFA. Pure fractions were collected and dried via lyophilization to yield the compound (302 mg, 49% yield). HRMS (ESI) calculated for C_9_H_11_N_3_^+^ is 161.1031, [M+H] found 162.1059. ^1^H NMR (500 MHz, CD_3_OH) δ 7.02 (d, *J* = 9.2 Hz, 1H), 6.79 (m, 2H), 4.58 (s, 1H), 2.79 (S, 6H). ^13^C NMR (151 MHz, CD_3_OH) δ_C_ 150.65, 133.01, 125.30, 123.23, 116.12, 94.64, 45.03

### Synthesis of methyl (*S*)-2-((*tert*-butoxycarbonyl)amino)-3-(4-((2-cyano-4-(dimethylamino)phenyl)amino) phenyl)propanoate (3).

2-amino-5-(dimethylamino)benzonitrile (**1**) (250 mg, 1.55 mmol, 1.5 equiv.) and methyl 2-((*tert*-butoxycarbonyl)amino)-3-(4-(((trifluoromethyl)sulfonyl)oxy)phenyl)propanoate (**2**) (440 mg, 1.03 mmol, 1 equiv.) were combined with BrettPhos Pd G1 (methyl *t*-Butyl ether adduct, 82 mg, 0.103 mmol, 0.1 equiv.) and cesium carbonate (1.010 g, 3.101 mmol, 3 equiv) in 8 mL of degassed dioxanes in a sealed round bottom flask. The flask was sparged and purged three times with argon, sealed and heated at 100 °C for roughly 24 hours. After the solution was cooled to room temperature, the contents were filtered through a short plug of silica gel using CH_2_Cl_2_ to transfer the material to the silica (10 mL), and then ethyl acetate (60 mL) was used to elute the product. The clarified solution was concentrated under reduced pressure and further purified by reverse phase chromatography (5% to 100% water in acetonitrile with 0.1% TFA). Pure fractions were collected and dried via lyophilization to yield the compound (256 mg, 46%). HRMS (ESI) calculated for C_24_H_30_N_4_O_4_^+^ is 438.2345, [M+H] found 439.2359. ^1^H NMR (500 MHz, CD_3_OH) δ 7.20 (d, *J* = 9.1 Hz, 1H), 7.10 – 6.98 (m, 3H), 6.93 (d, *J* = 3.0 Hz, 1H), 6.88 – 6.75 (m, 2H), 4.31 (t, *J* = 8.5, 5.7 Hz, 1H), 3.68 (s, 2H), 3.35 (s, 1H), 2.99 (dd, *J* = 13.8, 5.8 Hz, 1H), 2.92 (s, 6H), 2.83 (dd, *J* = 14.0, 8.6 Hz, 1H), 1.40 (s, 9H). ^13^C NMR (151 MHz, CDCl_3_)δC: 172.49, 155.12, 137.89, 133.24, 129.30, 127.90, 125.60, 123.24, 122.40, 121.35, 116.10, 115.92, 114.00, 99.03, 80.18, 54.57, 52.24, 37.53, 29.72

#### Synthesis of methyl (*S*)-2-((*tert*-butoxycarbonyl)amino)-3-(7-(dimethylamino)-9-oxo-9,10-dihydroacridin-2-yl)propanoate (Dad, 4):

A solution of 13.5 M (6 mL) sulfuric acid was added to a flask containing (*S*)-2-((*tert*-butoxycarbonyl)amino)-3-(4-((2-cyano-4-(dimethylamino)phenyl)amino)phenyl)propanoate (**3**) (250 mg, 0.570 mmol, 1 equiv.). The flask was refluxed at 115 °C with vigorous stirring for approximately 24 hours. The solution was cooled to room temperature and quenched using 25 mL of cold water, added dropwise. The pH was adjusted to 7 using 10 M NaOH. The resulting precipitate was collected using vacuum filtration and purified using reverse phase chromatography ((5% to 100% Water (0.1% TFA) in Acetonitrile (0.1% TFA)). Pure fractions were collected and dried via lyophilization to yield the desired product (96 mg, 52%). HRMS (ESI) calculated for C_18_H_19_N_3_O_3_^+^ is 325.1505, [M+H] found 326.1516. ^1^H NMR (500 MHz, CD_3_OH) δ_H_ 8.40 (s, 1H), 7.84 (dd, *J* = 8.8, 1.9 Hz, 1H), 7.82 – 7.74 (m, 3H), 7.35 (s, 1H), 4.39 (dd, *J* = 7.8, 6.1 Hz, 1H), 3.54 (dd, *J* = 14.7, 6.1 Hz, 1H), 3.38 (dd, *J* = 14.6, 7.8 Hz, 1H), 3.15 (s, 6H). ^13^C NMR (151 MHz, CD_3_OH) δ_C_ 170.93, 155.48, 147.91, 137.32, 135.25, 132.43, 131.07, 125.31, 123.89, 119.35, 119.00, 113.47, 111.10, 99.72, 54.79, 39.49, 36.31.

### Absorbance Measurements.

Dad was dissolved in MilliQ water and 100 uL of 2 M sodium hydroxide to make a to a 5 mM starting stock. Samples were individually diluted to the desired concentration using 50:50 acetonitrile to phosphate buffered saline (CH_3_CN/PBS) in triplicate. The absorbance spectrum was measured on a ThermoScientific Genesys 150 UV-Vis spectrometer, using 50:50 CH_3_CN/PBS as a blank. These absorbances were also measured using a Tecan M1000 plate reader with acridone as a standard.

### Fluorescence Measurements.

Fluorescence measurements were performed on 5 μM samples on either a Tecan M1000 plate reader or a Photon Technology International (PTI) QuantaMaster^™^ 40 fluorescence spectrometer. The pH dependent measurements were performed 50/50 CH_3_CN neutralized in alumina/citric acid BIS-TRIS propane (CBTP) buffer. The buffer system relies of varying ratios of citric acid to BIS-TRIS propane to achieve buffers with predictable pH’s. The pH for each buffer/CH_3_CN mixture was confirmed using a pH meter. Fluorescence lifetime measurements were collected using the QuantaMaster^™^ 40 TCSPC module with a 486 nm pulsed LED light source. QY measurements were performed using a Jasco FP-8300 fluorimeter with an ILF-835 integrating sphere attachment.

### Quantum Mechanical Calculations.

*Ab initio* electronic structure calculations of the absorption and emission spectra of Aad’ and Dad’ employed the APF-D density functional as implemented in the Gaussian16^™^ suite of programs with the 6–311+G(2d,p) basis set which has been recommended for calculations of fluorescence spectra.^29^ Using a previously described procedure,^30^ we combined Franck-Condon integral calculations with vibrational calculations of the ground and first excited states to generate vibronic spectra representing Aad’ absorption and emission spectra in aqueous solution. The differences in the lowest energy Aad’ and Dad’ vertical transitions were used to shift the Aad’ emission spectrum to approximate the Dad’ spectrum.

### Dad aaRS Activity Assay.

BL21-DE3 *E. coli* cells were transformed with a pBad plasmid encoding superfolder GFP with a TAG mutation at position 140 (sfGFP_TAG140_) and a pDule2 plasmid encoding one of three (G2, G11, or A9) previously evolved *Mj* tyrosine aaRSs.^13^ Cells were grown with ampicillin (Amp, 100 μg/mL) and streptomycin (Strep, 50 μg/mL) on an LB-agar plate. As a negative control, BL21-DE3 *E. coli* cells were transformed with only the sfGFP_TAG140_ plasmid, and grown with only ampicillin (Amp, 100 μg/mL) in parallel to the doubly transformed cells. Single colonies were picked and grown in non-inducing media^33^ (1 × 5 mL, 100 μg/mL Amp, 50 μg/mL) with shaking (250 RPM) at 37 °C until saturation. 5% of each primary culture was added to a 2 mL secondary culture in auto-inducing media^33^ that contained either 1 mM Acd (positive control), 1 mM Dad, or no fluorescent amino acid (negative control). Each secondary culture was split into 3×1 mL triplicate cultures and incubated with shaking (250 RPM) at 37 °C for 24 hours. Each culture was diluted 10-fold with MilliQ water and the sfGFP fluorescence intensity was quantified (λ_ex_: 488 nm, λ_em_: 509 nm). Additionally, the optical density was measured at 600 nM (OD_600_) to normalize the fluorescence reading to the number of cells.

### Dad Docking Model.

Using PyMol,^34^ the optimized ground state structure of Dad’ was aligned with the sidechain of Acd docked into a homology model of the G2 aaRS built using the crystal structure coordinates, as previously described.^13, 30–31^ The Dad’ molecule was aligned to position the dimethylamino group as in Dad.

## Supplementary Material

Supplementary Material

## Figures and Tables

**Scheme 1.**
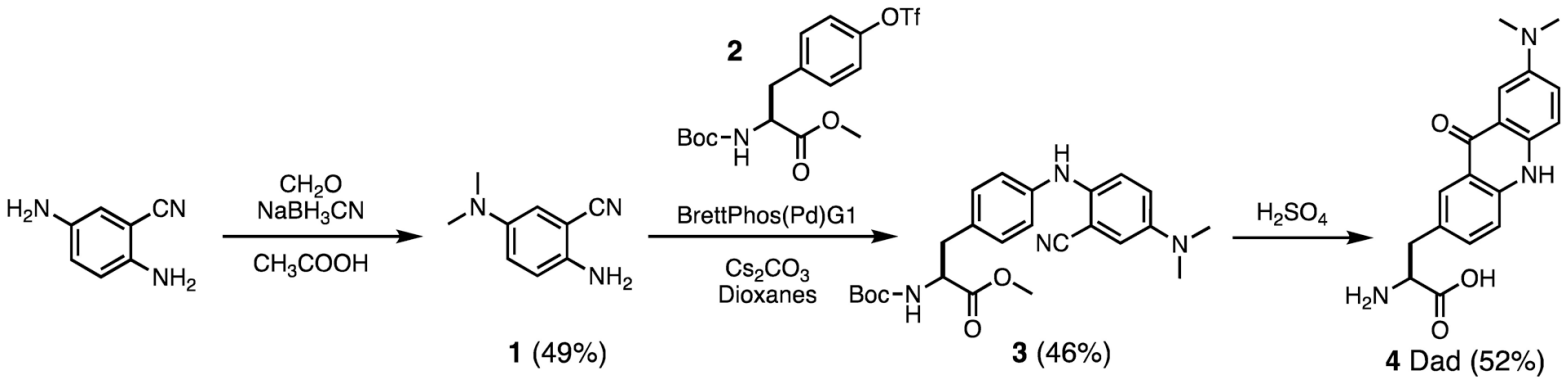
Synthesis of Dad.

**Table 1.** QY (unitless) of Aad and Dad in various solvents.*

	DMSO	MeOH	CH_3_CN	THF	PBS	CH_3_CN/PBS
Dad	0.57 ± 0.08	0.21 ± 0.04	0.24 ± 0.03	0.35 ± 0.03	0.06 ± 0.01	0.25 ± 0.02
Aad	0.12 ± 0.01	0.08 ± 0.01	0.09 ± 0.01	0.08 ± 0.04	0.05 ± 0.01	0.10 ± 0.02

* QY determined using an integrating sphere as described in Supplementary Material.

**Table 2.** Fluorescence lifetime (in ns) of Aad and Dad in various solvents.*

	DMSO	MeOH	CH_3_CN	THF	PBS	CH_3_CN/PBS
Dad	19.91 ± 0.02	12.64 ± 0.02	11.57 ± 0.02	12.85 ± 0.01	6.26 ± 0.01	12.92 ± 0.06
Aad	10.33 ± 0.06	10.83 ± 0.03	11.65 ± 0.03	11.59 ± 0.04	6.69 ± 0.02	11.82 ± 0.04

* Details of fitting including decay curves and residuals as well as chi-squared metrics are reported in Supplementary Material.
