# Supplementary material for: Synthesis and characterization of fluorescent amino acid dimethylaminoacridonylalanine
Source: ARKIVOC. Author manuscript; Available in PMC 2021 May 7. (PMC8104435; doi:10.24820/ark.5550190.p011.498)
Supplement: Supplementary Material [file NIHMS1697208-supplement-Supplementary_Material.pdf]

Supplementary Material

Synthesis and Characterization of Fluorescent Amino Acid Dimethylaminoacridonylalanine

Chloe M. Jones,[a,b] George A. Petersson,[c] and E. James Petersson[a]

[a]*Department of Chemistry; University of Pennsylvania; 231 South 34th Street; Philadelphia, Pennsylvania 19104-6323, USA*
[b]*Biochemistry and Molecular Biophysics Graduate Group; University of Pennsylvania; 3700 Hamilton Walk, Philadelphia, PA 19104, USA*
[c]*Temple University Institute for Computational Molecular Science, 1925 N. 12th Street, Philadelphia, PA 19122, USA*
Email: ejpetersson@sas.upenn.edu

Contents:

General information **S2**

Photophysical Properties of 7-(Dimethylamino)acridon-2-ylalanine (Dad) **S3**

Electronic Structure Analysis **S9**

Screening of Aminoacyl tRNA Synthetases for Dad Activity **S14**

NMR Spectra **S15**

References **S16**

General Information

Materials. *E. coli* BL21 DE3 cells were purchased from Agilent (Santa Clara, CA, USA). Milli-Q filtered (18 MΩ) water was used for all solutions (Millipore; Billerica, MA, USA). Methyl 2-((*tert*-butoxycarbonyl)amino)-3-(4-(((trifluoromethyl)sulfonyl) oxy)phenyl)propanoate was synthesized as previously described[1] using Boc-L-tyrosine methyl ester, purchased from ChemImpex (Wood Dale, IL, USA), and BrettPhos Pd G1 methyl *t*-butyl ether adduct, purchased from Sigma-Aldrich (St. Louis, MO, USA). All other reagents and solvents were purchased from Fisher Scientific (Pittsburgh, PA, USA) or Sigma-Aldrich unless otherwise specified.

Instruments. Low resolution electrospray ionization mass spectra (LRMS) were obtained on a Waters Acquity Ultra Performance LC connected to a single quadrupole detector (SQD) mass spectrometer (Milford, MA, USA). High resolution electrospray ionization mass spectra (HRMS) were collected with a Waters LCT Premier XE liquid chromatograph/mass spectrometer. Nuclear magnetic resonance (NMR) spectra were obtained on a Bruker DRX 500 MHz instrument (Billerica, MA, USA). Absorbance measurements were performed on a ThermoScientific Genesys 150 UV-Vis spectrometer (Waltham, MA, USA). Fluorescence readings fort the incorporation assay and molar absorptivity measurements were made on a Tecan M1000 plate reader (Mannedorf, Switzerland). Amino acid excitation and emission spectra and time correlated single photon counting (TCSPC) fluorescence lifetime measurements were made using a Photon Technology International (PTI) QuantaMaster™ 40 fluorescence spectrometer (currently, Horiba; Piscataway, NJ, USA). Quantum yield (QY) measurements were performed using a Jasco FP-8300 fluorimeter with an ILF-835 integrating sphere attachment (Easton, MD, USA).

Photophysical Properties of 7-(dimethylamino)acridon-2-ylalanine (Dad)

Molar Absorptivity Measurements. The core chromophore 2-(dimethylamino)acridone (Dad') was dissolved to a 2 mM starting stock in DMSO. Samples were individually diluted to the desired concentration using 50:50 CH_3CN/phosphate buffered saline (PBS) in triplicate. This solvent was chosen to mimic the conditions previously reported for corresponding measurements of 2-aminoacridone (Aad') and 7-aminoacridon-2-ylalanine (Aad).[2] The absorbance at 425 nm was measured on a Tecan M1000 plate reader. As standards to account for pathlength, the molar absorbtivities for acridone (420 nm) and Aad' (386 nm) were also measured. The molar absorptivity measurements were confirmed by measurement of a separate batch of Dad' and the values fitted to a line in Graphpad Prism to yield ε_{425} = 5415 ± 59 M^{-1}•cm^{-1}.

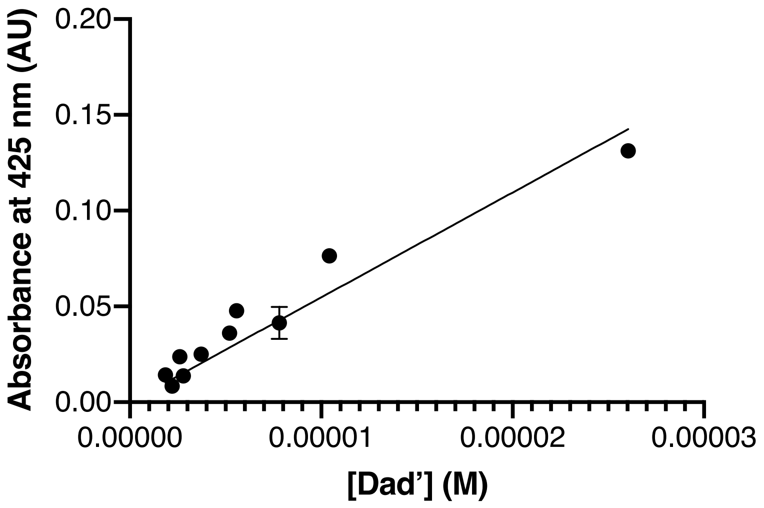

Figure S1: Molar absorptivity data for Dad' in 50:50 CH_3CN/PBS.

Absorption and Fluorescence Measurements. 7-(Dimethylamino)acridon-2-ylalanine (Dad) *a*bsorption measurements were performed either on a Tecan M1000 plate reader or a ThermoScientific Genesys 150 UV-Vis spectrometer. The pH dependent measurements were in Citric acid BIS-TRIS propane (CBTP) buffer. The buffer system relies of varying ratios of citric acid to BIS-TRIS propane to achieve buffers with predictable pH's. The buffer pH's were also confirmed using a pH meter. Fluorescence measurements were performed on 5 µM samples on either a Tecan M1000 plate reader or a Photon Technology International (PTI) QuantaMaster™ 40 fluorescence spectrometer.

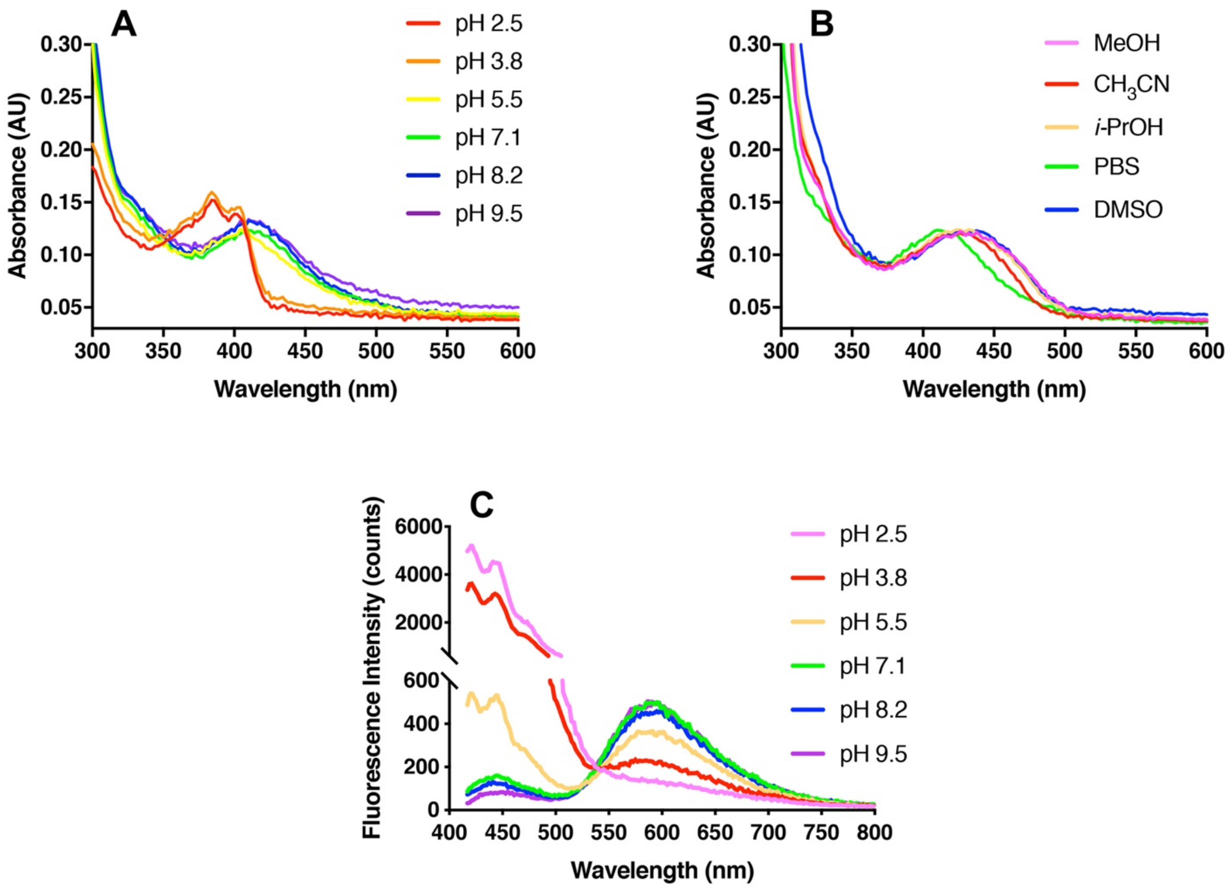

Figure S2. Absorption and emission spectra of Dad at various pHs and in various solvents. A) Absorption spectra in CBTP at various pHs, B) Absorption spectra in various solvents, C) Emission spectra in CH_3CN/CBTP at various pHs (λ_{Ex} = 425 nm).

Quantum Yield Measurements. The JASCO ILF-835 integrating sphere calculates quantum yield (QY) by comparing the integrated intensity of the incident (excitation) beam to the fluorescence emission of a sample. These values are compared in the presence and absence of a fluorophore. Within each replicate, the incident excitation light spectrum was collected in the presence of 2 mL of solvent. After measuring the incident light intensity, 5, 10, or 15 µL of a 5 mM dye stock was added to the solvent and the new excitation and emission spectra were collected.

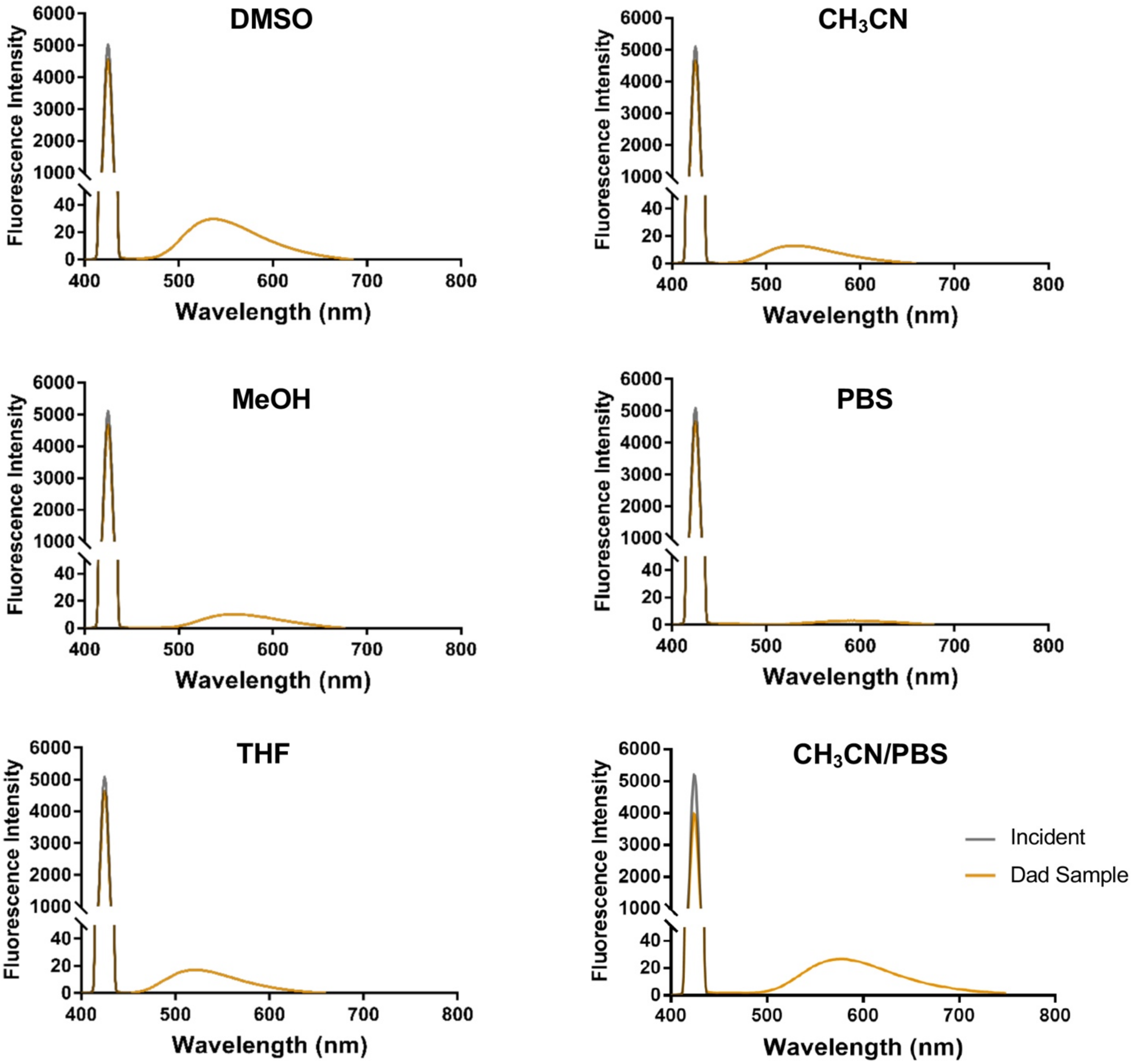

Figure S3. Representitive QY acquisitions for Dad in various solvents. All had identical excitation (425 nm, excitation bandwidth 5 nm) and spectral colection (400-800 nm, emission bandwidth 5 nm, detector sensitivity: low, scan speed: 1000nm/min) parameters.

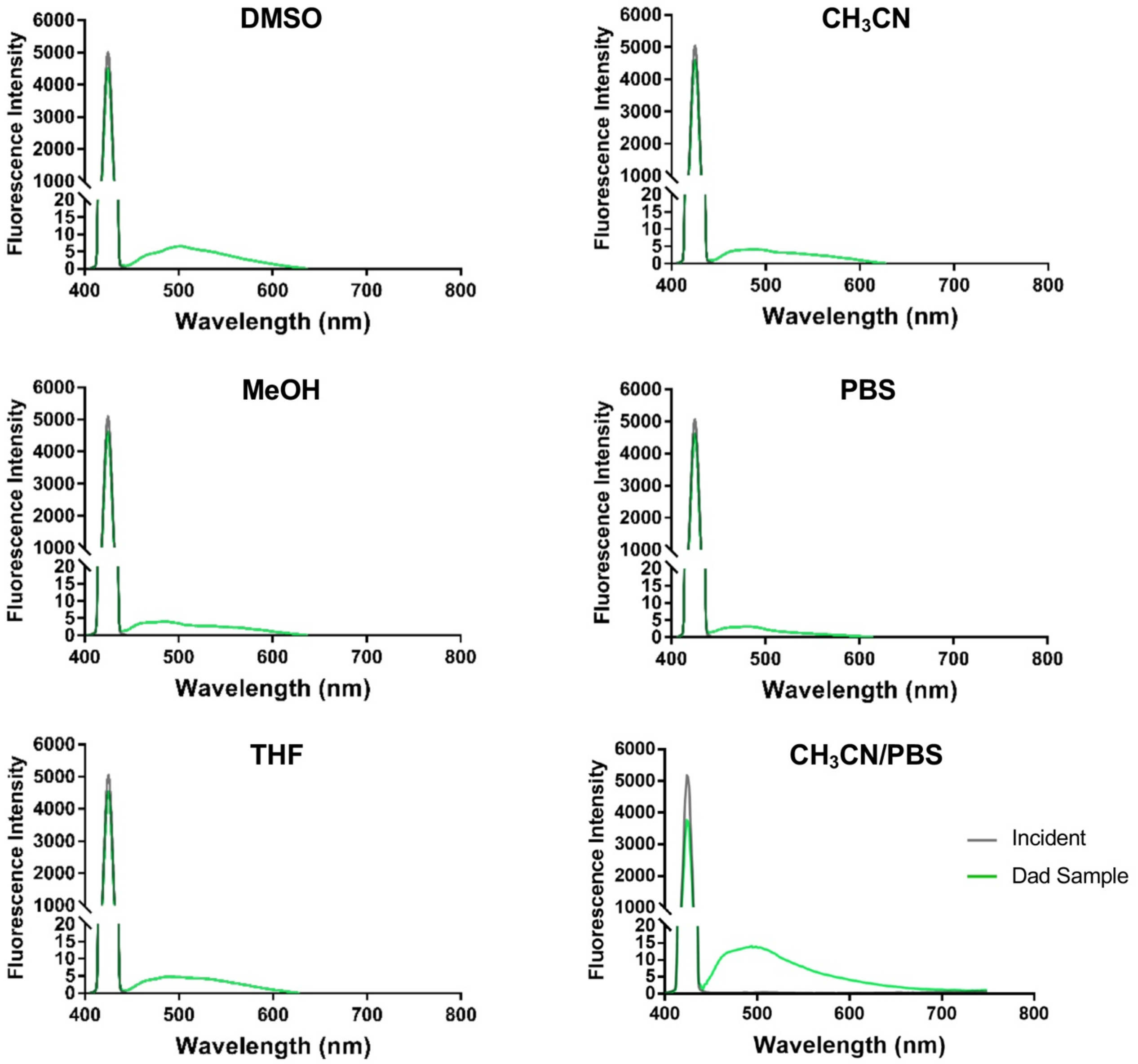

Figure S4. Representitive QY acquisitions for Aad in various solvents. All had identical excitation (425 nm, excitation bandwidth 5 nm) and spectral colection (400-800 nm, emission bandwidth 5 nm, detector sensitivity: low, scan speed: 1000nm/min) parameters.

Fluorescence lifetime measurements. TCSPC measurements of fluorescence lifetime decays for 5 µM samples of Dad and Aad were collected with the PTI Quantamaster™ 40 using a pulsed LED with a maximum emission at 486 nm and with a 480 nm short pass filter in the excitation beam path. Fluorescence emission was collected at 525 or 535 nm with 20 nm slit widths. The instrument response function (IRF) was collected under identical conditions. Data analysis was performed with FluoFit software (PicoQuant GmbH; Berlin, Germany) using an exponential decay model.

Table S1. Fluorescence lifetime (in ns) of Dad at concentrations in DMSO.

	10 µM	100 µM	1 mM
Dad	19.33 ± 0.03	19.61 ± 0.03	19.54 ± 0.03

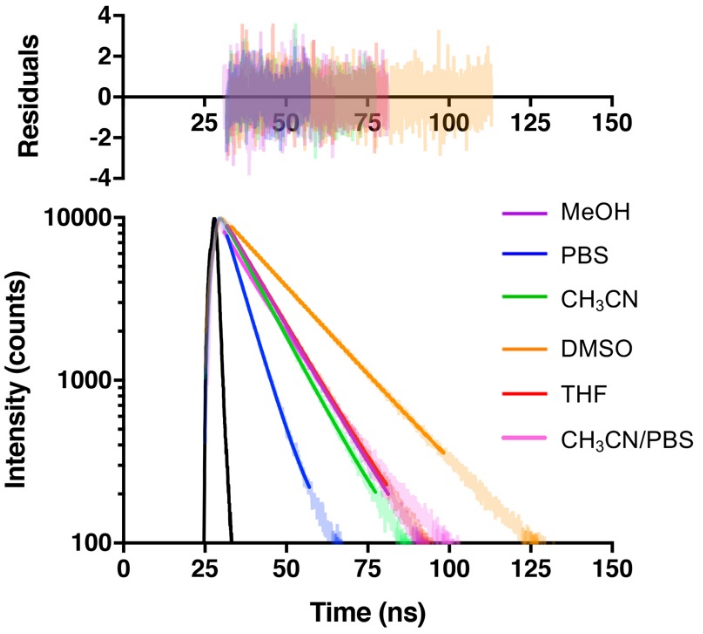

Figure S5. Fluorescence lifetime decays (bottom) and residuals after fitting (top) for Dad in various solvents. TCSPC decay curves (pale colors) collected with the emission monochromator set to 555 nm and fits to single exponential decay models (dark lines) are shown along with the corresponding residuals in like colors. All χ^2 values for fits ranged from 0.934 to 1.037.

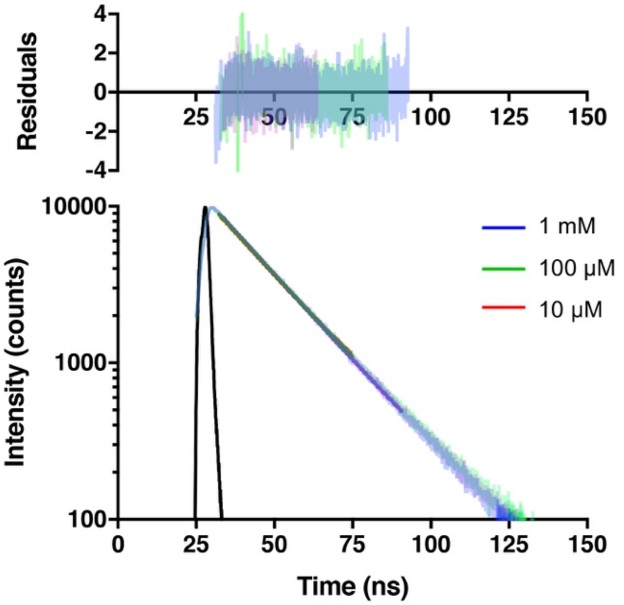

Figure S6. Fluorescence lifetime decays (bottom) and residuals after fitting (top) for Dad at various concentrations in DMSO. TCSPC decay curves (pale colors) collected with the emission monochromator set to 555 nm and fits to single exponential decay models (dark lines) are shown along with the corresponding residuals in like colors. All χ^2 values for fits ranged from 0.9996 to 1.334

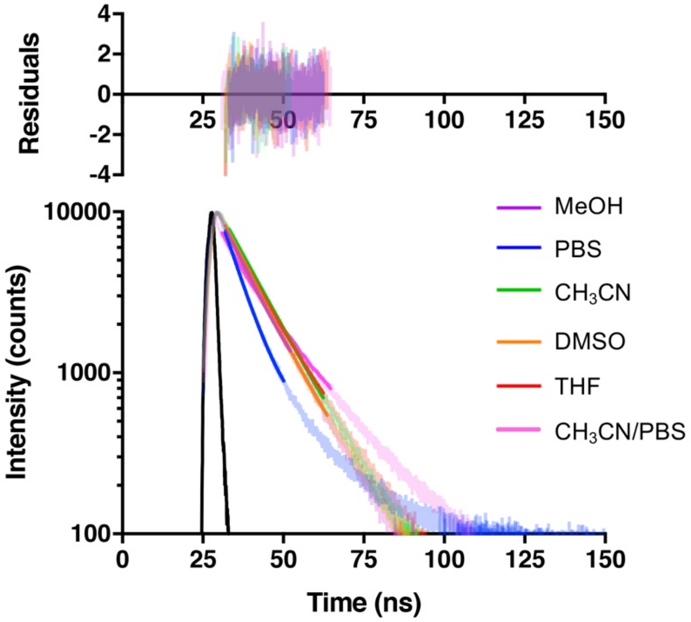

Figure S7. Fluorescence lifetime decays (bottom) and residuals after fitting (top) for Aad in various solvents. TCSPC decay curves (pale colors) collected with the emission monochromator set to 535 nm and fits to single exponential decay models (dark lines) are shown along with the corresponding residuals in like colors. All χ^2 values for fits ranged from 0.953 to 1.184.

Electronic Structure Analysis

Quantum Mechanical Calculations. *Ab initio* electronic structure calculations of the absorption and emission spectra for 2-aminoacridone (Aad') and 2-(dimethylamino)acridone (Dad') employed the APF-D density functional as implemented in the Gaussian16™ suite of programs with the 6-311+G(2d,p) basis set.[3] The APF-D/6-311+G(2d,p) optimized geometry input files for these calculations and the corresponding vertical transitions are provided on the following pages. We combined Franck-Condon integral calculations with vibrational calculations of the ground and first excited states [Gaussian16 keyword(options) Freq=(ReadFC,FranckCondon,ReadFCHT)] as described in Foresman and Frisch, pages 364 to 371, to generate a vibronic spectrum representing the Aad' emission spectrum in aqueous solution.[4] These spectra as well as the highest occupied molecular orbital (HOMO) and lowest unoccupied molecular orbital (LUMO) for Aad' are shown in **Figure S8**. We included the differential solvation of the ground state and first excited state by employing the procedure described in Foresman and Frisch, pages 371 to 379.[4] Unfortunately, the geometry changes between the ground and excited states for Dad' prevented completion of Franck-Condon calculations. Therefore, we approximated the Dad' solution emission spectrum by calculating the difference between the Dad' and Aad' vertical emissions ($\Delta\lambda_{Ex}$ = +22.58). We shifted the Aad' emission spectrum by these values to give the calculated spectrum shown in **Figure 4**.

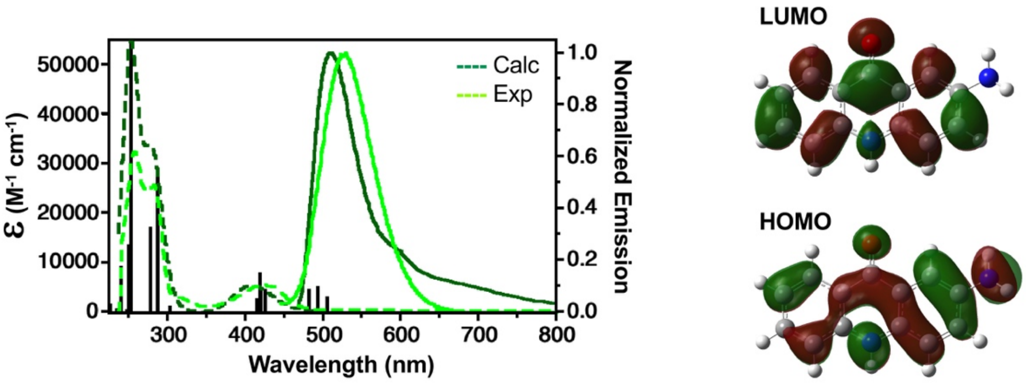

Figure S8. Comparison of experimental and computed Aad spectra. Experimental absorption and emission spectra for Aad were determined in CH_3CN/PBS. Computed spectra were determined from Aad' APF-D/6-311+G(2d,p) vertical excitation and emission calculations with Franck-Condon integral calculations to generate the vibronic emission spectrum. Excitation spectra are shown as dashed lines, emission spectra are shown as solid lines. Individual calculated state-to-state transitions are shown as black vertical lines.

2-(Dimethylamino)acridone (**Dad'**)

1-A Ground State in H2O

0 1

C	4.76928000	0.50018800	0.04628000
C	3.67894300	1.33823400	0.03006700
C	2.38069000	0.79695100	0.00816500
C	2.20350800	-0.60158400	0.00338100
C	3.33860900	-1.42819100	0.02006100
C	4.60663900	-0.89598000	0.04111000
C	-0.24750600	-0.23438600	-0.04207000
C	0.00666200	1.14455400	-0.03299500
C	-1.08108100	2.02807800	-0.03945300
H	-0.90456700	3.09919200	-0.01953400
C	-2.36894400	1.55256700	-0.06435200
C	-2.65618700	0.16217600	-0.09390400
C	-1.56603900	-0.70383100	-0.06379500
H	1.43997700	2.61631600	-0.00112000
H	5.76702300	0.92671000	0.06307000
H	3.80944800	2.41599400	0.03387300
H	3.17887300	-2.50089200	0.01559900
H	5.47630100	-1.54355900	0.05363800
H	-3.17697000	2.27295700	-0.06238000
H	-1.69299800	-1.77797800	-0.05843000
C	0.86448800	-1.18589600	-0.01855600
O	0.68119800	-2.40849200	-0.01787400
N	1.29379900	1.61743800	-0.00822800
N	-3.95914900	-0.29280000	-0.17135100
C	-5.03688100	0.60063000	0.20062100
H	-5.98385300	0.08084300	0.06544700
H	-4.96998400	0.93331900	1.24600800
H	-5.05666700	1.48326900	-0.44141700
C	-4.20123200	-1.70197100	0.04397200
H	-3.91668800	-2.03125200	1.05408500
H	-5.26107700	-1.90477100	-0.10077400
H	-3.64663600	-2.30391500	-0.67921100

Vertical Excitation Transitions

Wavelength (nm)	Oscillator Strength
436.21	0.0784
324.56	0.0003
316.23	0.0228
295.18	0.5344
287.62	0.0906
259.66	0.0857
255.50	0.6060
251.81	0.0482
246.90	0.1139
240.61	0.0680
236.71	0.0128
233.10	0.0008
224.92	0.0102
224.20	0.0280
222.96	0.0152
222.13	0.1083
217.72	0.0003
216.17	0.0082
213.28	0.0299
211.15	0.0004
205.65	0.0144
203.21	0.2800
202.29	0.0238
200.91	0.0865
200.77	0.0199

2-(Dimethylamino)acridone (**Dad'**)
1-A Excited State in H2O

0 1

C	4.78824400	0.51876500	0.00015500
C	3.65353600	1.34912500	0.00006700
C	2.38646800	0.78686400	-0.00005000
C	2.20609300	-0.61671200	-0.00003900
C	3.35434900	-1.42257200	0.00005700
C	4.63236400	-0.85764500	0.00014400
C	-0.24014000	-0.25073400	-0.00006100
C	-0.01592700	1.15005600	-0.00009400
C	-1.09916800	2.03891300	-0.00005000
H	-0.90573700	3.10731900	-0.00005100
C	-2.40552200	1.58088900	0.00000100
C	-2.65458900	0.18710900	0.00000800
C	-1.55011500	-0.70337600	-0.00002000
H	1.41297700	2.60339100	-0.00035600
H	5.77743600	0.96337200	0.00022900
H	3.76348500	2.42944100	0.00008200
H	3.21995400	-2.49823400	0.00005700
H	5.50540300	-1.50291300	0.00020800
H	-3.21533000	2.29641700	0.00002900
H	-1.69185500	-1.77578900	-0.00002800
C	0.87311800	-1.19183200	-0.00008400
O	0.67853600	-2.43617200	-0.00013000
N	1.26412000	1.60370500	-0.00019300
N	-3.92226300	-0.30428400	0.00008700
C	-5.05678800	0.59521000	0.00018300
H	-5.97395400	0.01466000	0.00047800
H	-5.04373100	1.23313800	0.88813400
H	-5.04411300	1.23285600	-0.88798100
C	-4.18129100	-1.73175400	-0.00002500
H	-3.75755900	-2.20498900	0.88868900
H	-5.25462800	-1.89419600	-0.00012600
H	-3.75742400	-2.20488300	-0.88872500

Vertical Emission Transitions

Wavelength (nm)	Oscillator Strength
499.79	0.0865
337.97	0.0000
328.79	0.0366
307.70	0.1290
304.26	0.4071
273.44	0.0138

2-Aminoacridone (**Aad'**)
1-A Ground State in H2O

0 1

C	3.32518000	0.15383800	-0.00439500
C	2.19351200	0.94904700	-0.00423700
C	0.90862700	0.39045400	-0.00526800
C	0.75655500	-1.00534600	-0.00327000
C	1.90200400	-1.81594000	-0.00131500
C	3.15243300	-1.24744100	-0.00393300
C	-0.26872700	1.25966300	-0.00358500
C	-1.63785300	-0.82630300	0.00049700
C	-1.56253100	0.58086100	-0.00049500
C	-2.75415000	1.32342900	0.00157500
H	-2.67190400	2.40472900	0.00084000
C	-3.98052600	0.70080900	0.00449800
C	-4.04177600	-0.70332300	0.00551600
C	-2.89345000	-1.46021800	0.00358000
H	2.27218600	2.03133300	-0.00366300
H	1.79813600	-2.89658100	0.00292900
H	4.02806800	-1.88998100	-0.00568600
H	-0.57204800	-2.57374600	0.00009800
H	-4.89479200	1.28383000	0.00609900
H	-5.00618500	-1.20093800	0.00795200
H	-2.94509600	-2.54456300	0.00459900
N	-0.49466900	-1.56717700	-0.00117300
N	4.60502900	0.69473800	-0.07099000
H	5.32917200	0.11571100	0.33013200
H	4.67707100	1.64531000	0.26307400
O	-0.17286500	2.49185000	-0.00453000

Vertical Excitation Transitions

Wavelength (nm)	Oscillator Strength
406.70	0.0784
325.35	0.0003
303.34	0.0228
281.14	0.5344
276.74	0.0906
252.89	0.0857
251.06	0.6060
247.17	0.0482
239.25	0.1139
234.35	0.0680
226.01	0.0128
225.14	0.0008
223.30	0.0102
215.24	0.0280
213.10	0.0152
211.73	0.1083
209.40	0.0003
208.16	0.0082
202.95	0.0299
202.27	0.0004
200.45	0.0144
406.70	0.2800
325.35	0.0238
303.34	0.0865
281.14	0.0199

2-Aminoacridone (**Aad'**)
1-A Excited State in H2O

C	-0.01041000	3.52527000	-1.25778000
C	-0.00811000	2.25445000	-1.86106000
C	-0.00116000	1.11713000	-1.06939000
C	0.00372000	1.20326000	0.34182000
C	0.00130000	2.48051000	0.91816000
C	-0.00571000	3.63051000	0.12214000
C	0.01098000	0.00078000	1.15549000
C	0.01289000	-1.27082000	0.43820000
C	0.00780000	-1.31108000	-0.98295000
C	0.00965000	-2.53750000	-1.66813000
H	0.00565000	-2.53657000	-2.75384000
C	0.01647000	-3.73671000	-0.97974000
C	0.02158000	-3.71443000	0.43037000
C	0.01969000	-2.47311000	1.12041000
H	-0.00247000	-0.17227000	-2.67138000
H	-0.01585000	4.41335000	-1.88008000
H	-0.01174000	2.15869000	-2.94265000
H	0.00502000	2.55077000	1.99953000
H	-0.00746000	4.60895000	0.59227000
H	0.01788000	-4.68175000	-1.51014000
H	0.02361000	-2.45286000	2.20507000
O	0.01545000	0.03919000	2.41200000
N	0.00112000	-0.13737000	-1.66098000
N	0.02828000	-4.85485000	1.14243000
H	0.03194000	-4.84048000	2.14971000
H	0.02985000	-5.75528000	0.69056000

Vertical Emission Transitions

Wavelength (nm)	Oscillator Strength
477.94	0.0947
337.96	0.0000
319.24	0.0409
301.57	0.0206
293.50	0.4467
265.82	0.0003

Screening of Aminoacyl tRNA Synthetases for Dad Activity. BL21-DE3 *E. coli* cells were transformed with a pBad plasmid encoding superfolder GFP (sfGFP) with a TAG mutation at position 140 ($sfGFP_{TAG140}$, Addgene #85483) and a pDule2 plasmid encoding one of three (G2, G11, or A9) previously evolved *Methanocaldococcus jannachii* (*Mj*) tyrosine aminoacyl tRNA synthetases (aaRSs).[5] Cells were grown with Acd, Dad or no unnatural amino acid (Uaa) added as described in the main text. Each culture was diluted 10-fold with MilliQ water and the sfGFP fluorescence intensity was quantified using a Tecan M1000 plate reader (λ_{ex}: 488 nm, λ_{em}: 509 nm). Additionally, the optical density was measured at 600 nM (OD_{600}) using a ThermoScientific Genesys 150 UV-Vis spectrometer. Raw data from triplicate experiments are shown in **Figure S9**. These were used to generate the graph of normalized fluorescence in **Figure 5**.

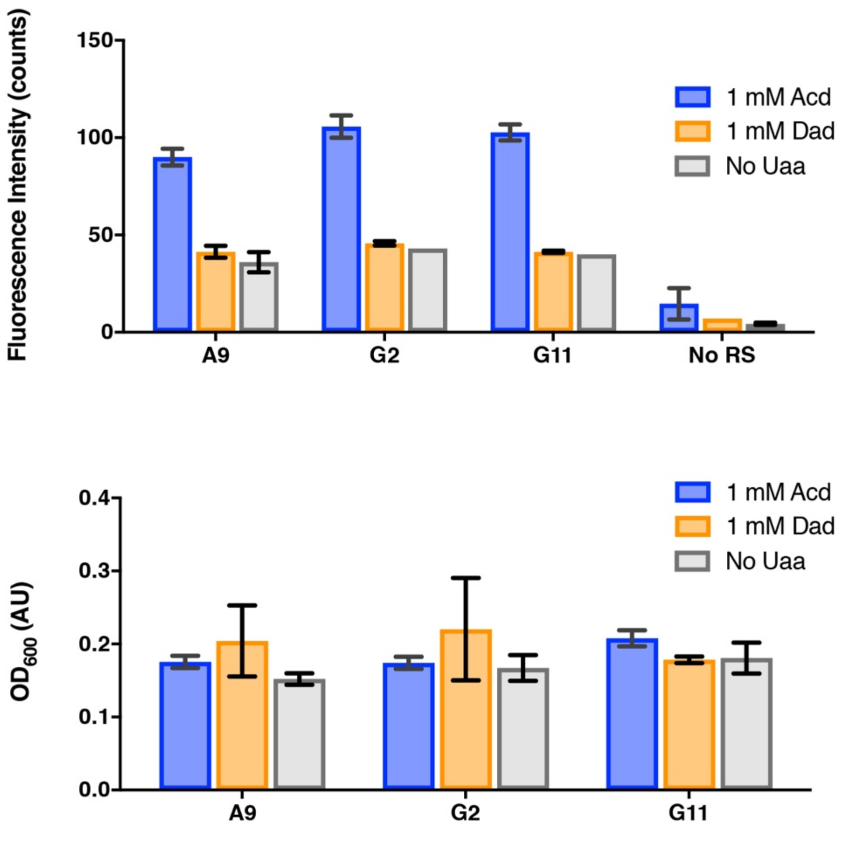

Figure S9. Comparison of sfGFP fluorescence intensity and cell density for cultures grown with three *Mj* aaRSs to investigate Dad incorporation ability.

NMR Spectra

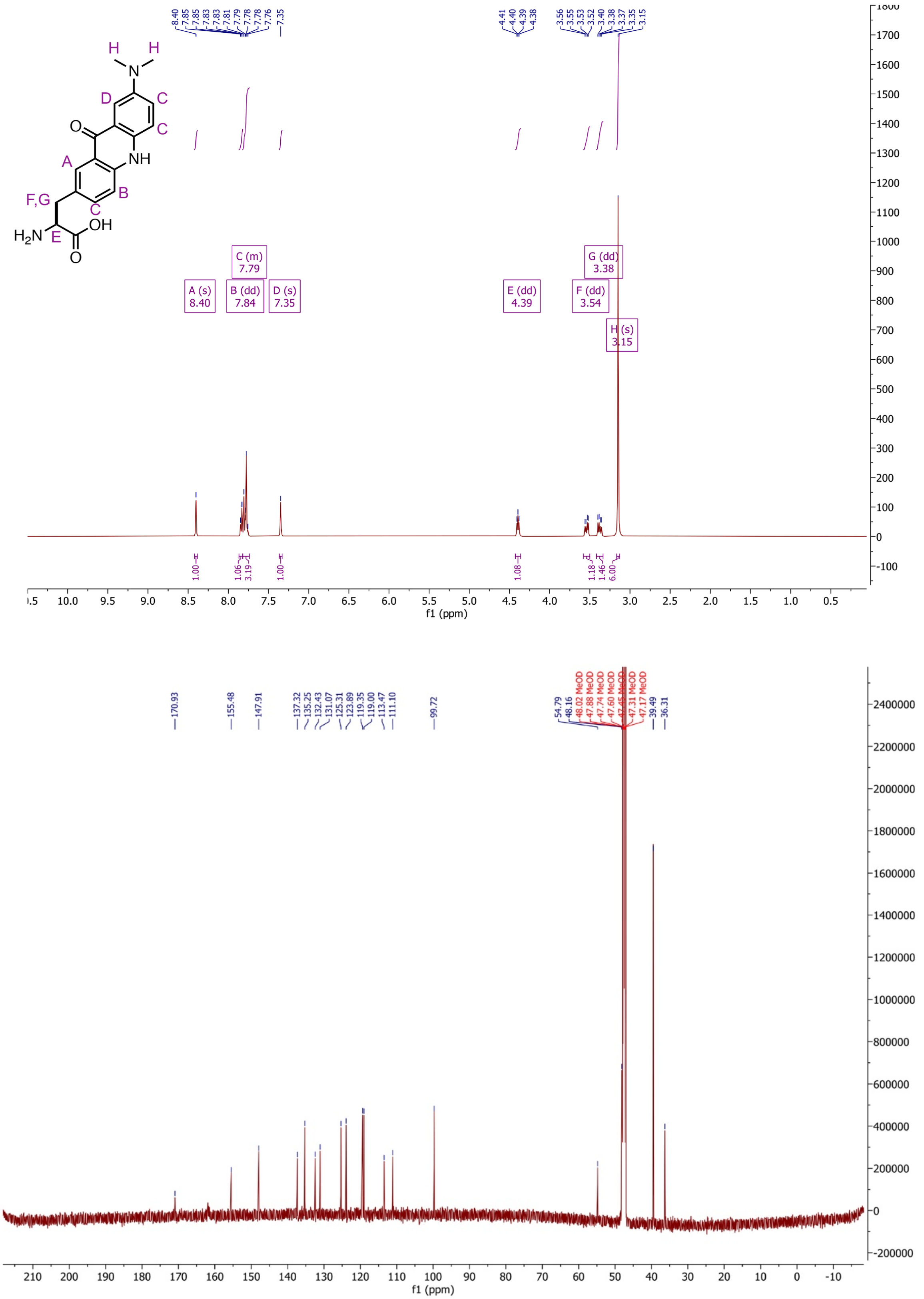

Figure 10. ^{1}H (Top) and ^{13}C (Bottom) NMR of **3** (Dad)

References

1. Jones, C. M.; Venkatesh, Y.; Petersson, E. J., Chapter Three - Protein Labeling for Fret with Methoxycoumarin and Acridonylalanine. In *Methods Enzymol.*, Chenoweth, D. M., Ed. Academic Press: 2020; Vol. 639, 37-69. https://doi.org/10.1016/bs.mie.2020.04.008

2. Sungwienwong, I.; Ferrie, J. J.; Jun, J. V.; Liu, C.; Barrett, T. M.; Hostetler, Z. M.; Ieda, N.; Hendricks, A.; Muthusamy, A. K.; Kohli, R. M.; Chenoweth, D. M.; Petersson, G. A.; Petersson, E. J., *J. Phys. Org. Chem.* **2018,** *31*, e3813. http://dx.doi.org/10.1002/poc.3813

3. Frisch, M. J.; Trucks, G. W.; Schlegel, H. B.; Scuseria, G. E.; Robb, M. A.; Cheeseman, J. R.; Scalmani, G.; Barone, V.; Petersson, G. A.; Nakatsuji, H.; Li, X.; Caricato, M.; Marenich, A. V.; Bloino, J.; Janesko, B. G.; Gomperts, R.; Mennucci, B.; Hratchian, H. P.; Ortiz, J. V.; Izmaylov, A. F.; Sonnenberg, J. L.; Williams-Young, D.; Ding, F.; Lipparini, F.; Egidi, F.; Goings, J.; Peng, B.; Petrone, A.; Henderson, T.; Ranasinghe, D.; Zakrzewski, V. G.; Gao, J.; Rega, N.; Zheng, G.; Liang, W.; Hada, M.; Ehara, M.; Toyota, K.; Fukuda, R.; Hasegawa, J.; Ishida, M.; Nakajima, T.; Honda, Y.; Kitao, O.; Nakai, H.; Vreven, T.; Throssell, K.; J. A. Montgomery, J.; Peralta, J. E.; Ogliaro, F.; Bearpark, M. J.; Heyd, J. J.; Brothers, E. N.; Kudin, K. N.; Staroverov, V. N.; Keith, T. A.; Kobayashi, R.; Normand, J.; Raghavachari, K.; Rendell, A. P.; Burant, J. C.; Iyengar, S. S.; Tomasi, J.; Cossi, M.; Millam, J. M.; Klene, M.; Adamo, C.; Cammi, R.; Ochterski, J. W.; Martin, R. L.; Morokuma, K.; Farkas, O.; Foresman, J. B.; Fox, D. J., Gaussian 16, Revision A.03. Gaussian, Inc.: Wallingford CT, 2016.

4. Foresman, J. B.; Frisch, Æ., *Exploring Chemistry with Electronic Structure Methods*. 3 ed.; Gaussian, Inc.: Wallingford, CT, 2015.

5. Sungwienwong, I.; Hostetler, Z. M.; Blizzard, R. J.; Porter, J. J.; Driggers, C. M.; Mbengi, L. Z.; Villegas, J. A.; Speight, L. C.; Saven, J. G.; Perona, J. J.; Kohli, R. M.; Mehl, R. A.; Petersson, E. J., *Org. Biomol. Chem.* **2017,** *15*, 3603-3610. http://dx.doi.org/10.1039/C7OB00582B
